# Kidney REPLACEment therapies in patients with acute kidney injury and RHABDOmyolysis (ReplaceRhabdo): a pilot trial

**DOI:** 10.1186/s12882-025-03945-3

**Published:** 2025-01-14

**Authors:** Lorenz Weidhase, Antonia Borrmann, Anja Willenberg, Meinhard Mende, Christina Scharf-Janßen, Sirak Petros, Jonathan de Fallois

**Affiliations:** 1 Medical Intensive Care Unit, University Hospital Leipzig, Leipzig, Germany; 2 Institute for Laboratory Medicine, Clinical Chemistry and Molecular Diagnostics, University Hospital Leipzig, Leipzig, Germany; 3 Institute for Medical Informatics, Statistics Und Epidemiology, University Leipzig, Leipzig, Germany; 4 Department of Anesthesiology, University Hospital, LMU Munich, Munich, Germany; 5 Medical Department III, Division of Nephrology, University Hospital Leipzig, Leipzig, Germany

**Keywords:** Myoglobin clearance, Rhabdomyolysis, Acute kidney injury (AKI), High cut-off dialyzer, Adsorber, CytoSorb, Continuous veno-venous hemodialysis (CVVHD), Continuous veno-venous hemofitration (CVVH)

## Abstract

**Background:**

Rhabdomyolysis is frequently associated with acute kidney injury (AKI). Due to the nephrotoxic properties of myoglobin, its rapid removal is relevant. If kidney replacement therapy (KRT) is necessary for AKI, a procedure with effective myoglobin elimination should be preferred. This pilot trial was designed to compare different KRT modes that enable myoglobin elimination.

**Methods:**

In this prospective randomized single-center study, 15 patients with rhabdomyolysis and severe AKI requiring KRT were randomized 1:1:1 into three groups: continuous veno-venous hemofiltration (CVVH), continuous veno-venous hemodialysis (CVVHD) using a high cut-off dialyzer (CVVHD-HCO), or CVVHD using a high-flux dialyzer in combination with the adsorber CytoSorb (CVVHD-CS). Concentrations of serum myoglobin, urea, creatinine, β2-microglobulin, interleukin-6, and albumin were measured before and after the dialyzer 1, 6, 12, and 24 h after initiating KRT.

**Results:**

There was no significant difference in the median myoglobin clearance between the KRT modes during the 24-h study period. Nevertheless, the CVVHD-CS group showed a significantly higher myoglobin elimination compared to the other modes in the first hours of treatment. However, as a greater decline in clearance performance was observed over time, no better performance was detected over the whole study period. Simulation of different device combinations showed the highest myoglobin clearance for CVVHD-HCO combined with CS with a 12-hourly adsorber exchange interval.

**Conclusions:**

All tested modes showed an effective myoglobin elimination capacity. The time-dependent elimination performance could be further increased by combining KRT with more frequent adsorber exchange.

**Trial registration:**

German Clinical Trials Registry (DRKS00023998); date of registration 03/03/2021.

**Supplementary Information:**

The online version contains supplementary material available at 10.1186/s12882-025-03945-3.

## Background

The association between crush syndrome following trauma and acute kidney injury (AKI) was first described in 1941 with muscle necrosis identified as the cause [1]. In addition to trauma, muscle hypoxia and overexertion, infections, autoimmune diseases, metabolic and electrolyte disorders, poisoning, drugs, hypo- and hyperthermia as well as genetic defects can trigger rhabdomyolysis (RM) [2–4]. Damaged skeletal muscle cells release intracellular contents, including myoglobin. These in turn result in damage to the kidneys by several mechanisms: Firstly, the formation of free radicals increases the oxidative stress of the renal tubular epithelial cells. Secondly, vasoconstriction due to cytokine release from the damaged muscles and volume depletion after trauma lead to renal hypoperfusion. Thirdly, myoglobin forms casts that obstruct the renal tubules and collecting ducts [2, 4–6].

About 5–25% of AKI cases are associated with RM [2, 5]. Harmful serum myoglobin levels correlate with the severity of AKI, with high mortality rates reported in patients with RM [7–9]. Therefore, rapid removal of myoglobin from the circulation can be useful in terms of preventing kidney damage.

If kidney function is preserved, myoglobin can be eliminated with forced diuresis and urin alkalinization [3, 4]. However, high-volume fluid therapy also appears to be unfavorable [10]. In oligo-anuric AKI, serum myoglobin can only be eliminated with kidney replacement therapy (KRT). Since myoglobin has a molecular weight of 17.1 kDa, sufficient middle molecule clearance is required. Effective myoglobin clearance was demonstrated with continuous veno-venous hemofiltration (CVVH) [11]. Subsequent animal experiments showed that early CVVH can reduce renal damage in RM [12]. Accordingly, this mode has been suggested as the method of choice [2, 4].

Newer KRT modes that enable effective myoglobin elimination, such as continuous veno-venous hemodialysis (CVVHD) using a high cut-off (HCO) dialyzer [13–16] and CVVHD in combination with an adsorber, have been investigated during the last years [15–19].

Based on these observations regarding the impact of myoglobin on renal function and the various modes available for its clearance, this pilot study aimed to compare the myoglobin elimination performance of different KRT modes. For this purpose, CVVH, CVVHD-HCO and CVVHD-CS were compared in patients suffering from RM and requiring KRT for severe AKI.

## Methods

### Study design

This study is a prospective, randomized, single-blinded and single-center pilot trial. It was registered at the German trial registry (DRKS00023998) and approved by the local ethics committee (ethics committee at the medical faculty, University Leipzig, 361/20-ek) and adheres to CONSORT guidelines (Additional file 1).

We planned to enroll 15 critically ill adult medical patients managed in the medical intensive care unit (ICU) of the University Hospital Leipzig. A written informed consent was obtained from all patients or their legal guardians.

Allocation concealment and unrestricted randomization were guaranteed by using 15 sequentially numbered, opaque sealed envelopes to avoid the possibility to decipher the allocation arm, with five patients planned for each intervention arm. The 15 envelopes were then thoroughly mixed and marked with unique numbers, and then kept in a container [20]. For technical reasons, only patients were blinded to the treatment arm. Enrollment of participants, randomization and assignment to interventions were carried out by trained medical staff.

The study received funding from CytoSorbents Europe, 12587 Berlin, Germany.

### Patients

Patients with severe AKI and indication for KRT (based on the recommendations of the Kidney Disease, Improving Global Outcomes [21]) as well as RM with serum myoglobin > 4000 µg/l were included. Exclusion criteria were refusal of study participation, participation in other trials, age < 18 years, pregnancy and lactation, futility of ICU treatment due to end-stage disease or expected death within the next 24 h, preexisting chronic kidney disease with glomerular filtration rate < 15 ml/min/1.73m^2^ (CKD G5), contraindications for systemic anticoagulation with unfractionated heparin.

All patients admitted to the ICU during the study period were screened for eligibility. The screening ended after the inclusion of 15 patients randomized to one of the following treatment groups:

- CVVH with high-flux dialyzer
- CVVHD with HCO dialyzer
- CVVHD with high-flux dialyzer and additional adsorber CytoSorb®

CVVH with high-flux dialyzer was defined as the control mode, because this has been the traditional procedure of choice in the literature [2, 4].

### Data collection

Demographic and clinical data were collected at the time of initiating KRT. These included age, gender, height, weight, body mass index (BMI), diuresis, presence of AKI stage 3, serum creatinine and estimated glomerular filtration rate according the CKD-EPI equation [22], serum urea, blood gas analysis, serum electrolytes, hemoglobin, platelet and leucocyte count, creatinine kinase, myoglobin, Acute Physiology And Chronic Health Evaluation (APACHE)-II score [23]), Sequential Organ Failure Assessment (SOFA) score [24], mean arterial blood pressure, need for mechanical ventilation, need for vasopressors, diagnosis of sepsis according to the sepsis-3 criteria [25], comorbidities, causes of rhabdomyolysis and indications for KRT.

Outcome and safety parameters were taken from electronic patient records or generated through telephone calls to patients, legal representatives or managing physicians.

### Procedure

A 13 French double-lumen high-flow catheter (Achim Schulz-Lauterbach VMP, Iserloh, Germany) was inserted as a central venous access for KRT. All KRT modes were performed using MultiFiltrate® with bicarbonate-buffered replacement fluid (multiBic® K4 or K2) or dialysate (CiCa® dialysate K4 or K2) (both: Fresenius medical care, Bad Homburg, Germany). The following dialysers were used: In CVVH, postdilution with a high-flux dialyzer (Ultraflux AV1000S; Fresenius Medical Care, Bad Homburg, Germany), in CVVHD-HCO the high-cut-off dialyzer (Ultraflux EMiC2, Fresenius Medical Care, Bad Homburg, Germany), and in CVVHD-CS, the same high-flux dialyzer together with the adsorber CytoSorb® (CytoSorbents Europe GmbH, Berlin, Germany). The adsorber was integrated into the extracorporeal circuit before the dialyzer. The anticoagulation of the extracorporeal circuit was performed in CVVHD-HCO and CVVHD-CS with regional citrate anticoagulation (RCA) and in CVVH using systemic anticoagulation with unfractionated heparin. Heparin was started at a dose of 18 IU/kg/h (ideal body weight) and adjusted during the treatment period based on the activated partial thromboplastin time (aPTT) monitored twice daily. The target aPTT was set at 60 s. RCA in CVVHD-HCO and CVVHD-CS was monitored by measuring ionized postfilter calcium and systemic ionized calcium every 6 h. Citrate supply (citrate: 136 mmol/l) was started with a citrate flow of 4.0 mmol citrate/l blood to achieve an ionized postfilter calcium concentration of 0.25–0.34 mmol/l. A calcium chloride solution (calcium: 83 mmol/l) was added simultaneously to the extracorporeal circuit near the backflow to the patient to keep systemic ionized calcium stable between 1.12–1.20 mmol/l. The calcium flow was started with 1.7 mmol/l Ca^2+^/l dialysate.

The total turnover rate (TTR) (dialysate or replacement fluid) was calculated at 25 ml/kg ideal body weight or, in obese patients, adjusted body weight/h [26]. We applied the Hamwi equation to calculate the ideal body weight (for males: 48 kg for the first 152 cm + 1.1 kg for each additional cm; for females 45 kg for the first 152 cm + 0.9 kg for each additional cm). The adjusted body weight was used for calculation of dialysate or replacement fluid flow if the quotient of actual body weight divided by ideal weight was more than 1.3: for males: (actual body weight-ideal body weight) * 0.38 + ideal body weight; for females: (actual body weight-ideal body weight) * 0.32 + ideal body weight) [27, 28].

In patients with CVVHD, the blood flow (*Qb*) was set to be three times higher than the dialysate flow. CVVH was adjusted to filter a maximum of 20% of the plasma water.

Ultrafiltration was set at zero ten minutes before collecting blood samples to avoid additional effect of hemoconcentration at the dialyzer. Blood samples were taken before (pre) and after (post) the dialyzer in patients with CVVH and CVVHD-HCO. In patients with CVVHD-CS, blood samples were collected before the adsorber (pre), between the adsorber and dialyzer (int) and after (post) the dialyzer.

Dialyzer and adsorber were exchanged according to the specifications of the manufacturer regarding the maximum lifespan (maximum dialyzer lifespan limited to 72 h and adsorber lifespan to 24 h). After the study-related interventions, the decision to continue or discontinue KRT was left to the discretion of the treating physician.

### Endpoints

The primary endpoint was defined as the difference of marginal means in myoglobin clearance from an ANOVA model with repeated measurements using CVVH as reference category. We used estimated marginal means taking into account the unbalanced dataset resulting from the small sample size and repeated measurements at different time points. Secondary endpoints of the study were: 1. myoglobin clearance after 1 h, 6 h, 12 h, and 24 h; 2. clearance of other molecules with different molecular weights (urea, creatinine, β2-microglobulin, interleukin-6 (IL-6), and albumin); 3. lifetime of the extracorporeal circuits. Adverse events (AEs) and serious adverse events (SAEs) were systematically recorded up to 72 h after the start of the study intervention. Furthermore, ICU length of stay, ICU mortality, hospital mortality as well as mortality at day 28 and day 90 were presented.

### Calculations

The calculations have been adapted as already described [13, 14] and are listed in detail under Additional file 2.

### Laboratory analyses

All biochemical analyses were performed at the Institute of Laboratory Medicine, Clinical Chemistry and Molecular Diagnostics, Leipzig University Hospital. Serum samples were analyzed shortly after blood withdrawal on a Cobas 8000 automated laboratory analyzer (Roche Diagnostics, Mannheim, Germany) in accordance with the manufacturer's protocol. The exact methods used in the field can be found under Additional file 3.

Laboratory samples with serum myoglobin concentrations above the measurement limit of 30,000 µg/l were diluted 1:20 as well as 1:50 and measured again. The concentration accurately measured with the lowest dilution was used for further analysis.

### Statistical analyses

This study was designed as a pilot trial, as a sample size calculation was not possible due to lack of appropriate published data. Therefore, the aims of the study were primarily exploratory.

Categorical variables were displayed as frequencies with percentages and tested with the Chi-square test (two-sided) or Fisher exact test as appropriate. Because of the small number of cases in the three groups and generally unreliable tests for normal distribution for such small sample sizes, continuous variables were presented as median with 25th and 75th quantiles in square brackets. Group comparisons were performed with the Kruskal–Wallis test. Since a combination of adsorber and high-flux dialyzer was used in CVVHD-CS, we compared in detail the substance-specific clearances between adsorber and dialyzer (Cl_int_) as well as after both (Cl_post_) using Wilcoxon signed-rank test. By adding up the medians of myoglobin clearances, models for the combination of different modes and more frequent adsorber exchange were developed.

Marginal means and their differences were displayed with 95% confidence interval and calculated by a mixed linear model for comparisons of clearance between the different KRT modalities using the CVVH as the reference mode. Dialyzer performance in one group over time has been analyzed by a general linear model with repeated measurements. The Kaplan–Meier estimate was applied to calculate and depict the survival function of the dialyzer lifetime. All analyses were performed using IBM SPSS, version 29 (Minneapolis, USA). The significance level was set at *p* < 0.05 for two-sided tests.

## Results

### Patients

**Fig. 1:**
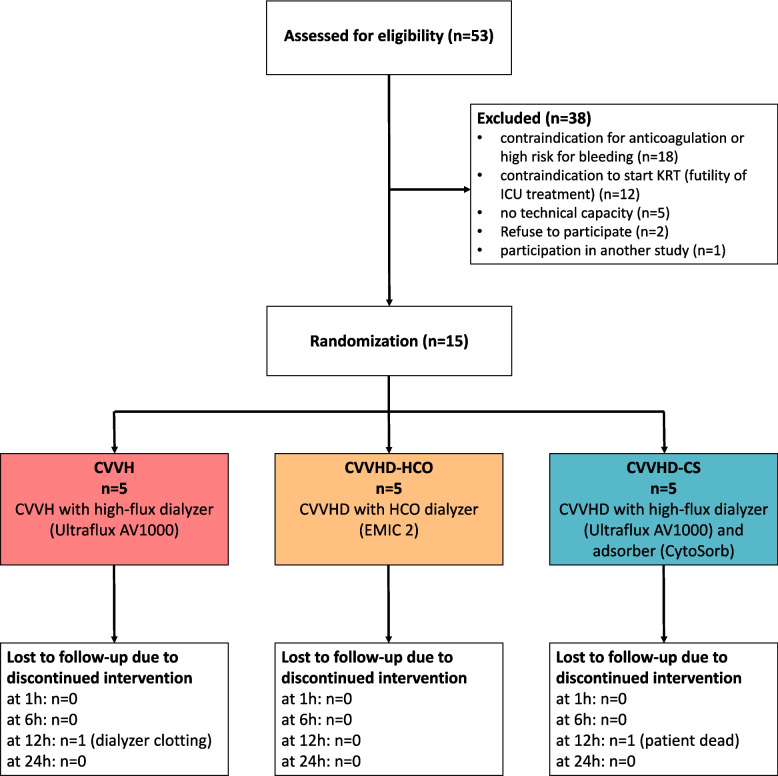
Recruitment flowchart AV1000S type of high-flux dialyzer, CVVH continuous veno-venous hemofiltration, CVVHD continuous veno-venous hemodialysis, EMiC2 type of high cut-off dialyzer, HCO high cut-off

A total of 53 patients were screened between April 2021 and December 2023. The main reasons for exclusion were contraindication for heparin anticoagulation related to bleeding (*n* = 18) and futility of intensive care treatment because of moribund patients (*n* = 12). Finally, 15 patients could be included in the study (Fig. 1). The baseline clinical characteristics, comorbidities, causes of RM and the indications for KRT did not differ between the three groups (Table 1). The initial laboratory parameters at the time of diagnosis were also similar (Table 2).

**Table 1:** Patient characteristics of study population

Parameter	All *n* = 15	CVVH *n* = 5	CVVHD-HCO *n* = 5	CVVHD-CS *n* = 5	*p*
**General characteristics:**					
Age (years)	68 [62; 77]	62 [47; 72]	74 [66; 77]	75 [61; 81]	0.223
Sex (male) n, (%)	12 (80.0)	5 (100.0)	4 (80.0)	3 (60.0)	0.287
High (cm)	175 [169; 180]	180 [175; 182]	175 [167; 180]	172 [160; 178]	0.200
Weight (kg)	94 [82; 108]	102 [91; 150]	94 [80; 104]	82 [72; 96]	0.129
BMI (kg/m^2^)	31.6 [26.8; 34.5]	34,5 [28.4; 45.3]	32.2 [27.0; 33.0]	27.7 [25.2; 33.4]	0.289
Mechanical ventilation n, (%)	9 (60)	4 (80)	2 (40)	3 (60)	0.435
Sepsis n, (%)	7 (47)	2 (40)	3 (60)	2 (40)	0.765
Vasopressors n, (%)	10 (67)	4 (80)	3 (60)	3 (60)	0.741
AKI 3 n, (%)	13 (87)	4 (80)	4 (80)	5 (100)	0.562
SOFA	13 [11; 15]	12 [10; 17]	12 [10; 14]	14 [12; 16]	0.586
APACHE II	31 [28; 39]	29 [28; 39]	30 [29; 35]	42 [30; 44]	0.277
MAP (mmHg)	65 [61; 72]	65 [59; 76]	65 [61; 74]	62 [59; 68]	0.618
24-h diuresis (ml)^a^	180 [68; 433]	215 [43; 508]	100 [45; 455]	260 [45; 570]	0.997
**Comorbidities:**					
• Chronic heart failure (NYHA IV) n, (%)	0 (0)	0 (0)	0 (0)	0 (0)	
• Pre-existing immunosuppression n, (%)	1 (7)	1 (20)	0 (0)	0 (0)	0.343
• Liver cirrhosis n, (%)	1 (7)	1 (20)	0 (0)	0 (0)	0.343
• Active malignancy n, (%)	2 (13)	1 (20)	0 (0)	1 (20)	0.562
• Chronic pulmonary disease n, (%)	0 (0)	0 (0)	0 (0)	0 (0)	
• eGFR < 45 ml/min/1.73m^2^ n, (%)	2 (13)	1 (20)	1 (20)	0 (0)	0.562
**Causes of rhabdomyolysis:**					
• Muscle hypoxia n, (%)	6 (40)	1 (20)	2 (40)	3 (60)	
• Drugs and toxins n, (%)	1 (7)	1 (20)	0 (0)	0 (0)	
• Shock and post CPR n, (%)	8 (53)	3 (60)	3(60)	2 (40)	0.517
**Indications for KRT:**					
• Acidosis (pH < 7,25) n, (%)	6 (40)	2 (40)	3 (60)	1 (20)	0.435
• High potassium level (> 6 mmol/l) n, (%)	2 (13)	2 (40)	0 (0)	0 (0)	0.099
• Pulmonary edema n, (%)	6 (40)	2 (40)	2 (40)	2 (40)	1.000
• Uremia (urea > 25 mmol/l) n, (%)	6 (40)	2 (40)	3 (60)	1 (20)	0.435
• Oliguria/Anuria n, (%)	11 (73)	3 (60)	5 (100)	3 (60)	0.256

Data presented as *n* (%) or median [25th, 75th quantile]

*AKI* acute kidney injury, *APACHE II* Acute Physiology And Chronic Health Evaluation II**,** *BMI* body mass index, *CPR* cardiopulmonary resuscitation, *CytoSorb®, CVVH* continuous veno-venous hemofiltration, *CVVHD* continuous veno-venous hemodialysis, *GFR* glomerular filtration rate, *HCO* high cut-off, *HR* heart rate, *KRT* kidney replacement therapy, *MAP* mean arterial pressure, *NYHA* New York Heart Association, *SOFA* sequential organ failure assessment

^a^24-h-diuresis before initiation of KRT

**Table 2:** Laboratory parameters at diagnosis

Parameter	All *n* = 15	CVVH *n* = 5	CVVHD-HCO *n* = 5	CVVHD-CS *n* = 5	*p*
Myoglobin (µg/l)	9791 [6522; 27931]	17560 [8157; 23816]	7692 [6434; 19938]	7773 [4821; 28966]	0.689
CK (µkat/l)	133 [44; 367]	212 [132; 344]	68 [47; 254]	37 [20; 367]	0.497
Urea (mmol/l)	17.0 [12.0; 28.0]	23.0 [11.5; 32.5]	19.0 [10.5; 27.5]	16.0 [12.0; 22.5]	0.733
Creatinine (µmol/l)	296 [179; 362]	296 [228; 351]	362 [132; 441]	275 [166; 659]	0.925
Phosphate (mmol/l)	1.98 [0.96; 2.68]	2.29 [1.11; 3.62]	1.15 [0.91; 2.44]	1.82 [1.03; 3.28]	0.659
Calcium (mmol/l)	1.95 [1.73; 2.2]	1.9 [1.77; 1.97]	2.07 [1.82; 2.33]	1.79 [1.70; 2.23]	0.589
Magnesium (mmol/l)	1.05 [0.95; 1.15]	1.15 [1.06; 1,27]	0.99 [0.79; 1.1]	1.04 [0.94; 1.13]	0.121
Sodium (mmol/l)	139.0 [132.0; 142.0]	137.0 [131.5; 143.0]	139.0 [138.5; 141.5]	141.0 [132.0; 144.5]	0.488
Potassium (mmol/l)	4.7 [4.6; 5.3]	5.2 [4.7; 5.6]	4.6 [4.1; 5.2]	4.7 [4.35; 5.1]	0.247
pH	7.32 [7.31; 7.41]	7.32 [7.27; 7.34]	7.39 [7.25; 7.42]	7.32 [7.27; 7.42]	0.481
Bicarbonate (mmol/l)	19.7 [16.7; 27.7]	16.7 [15.3; 30.4]	22.0 [17.75; 31.0]	19.7 [16.5; 26.8]	0.651
Base excess (mmol(l)	-4.1 [-9.7; 3.3]	-8.8 [-10.05; 3.85]	-3.3 [-7.15; 6.3]	-4.1 [-8.85; 1.8]	0.651
Lactate (mmol/l)	2.2 [1.5; 2.5]	2.4 [2.25; 2.85]	2.1 [1.0; 2.35]	1.6 [1.25; 3.6]	0.295
Hemoglobin (mmol/l)	6.0 [4.4; 6.9]	6.9 [5.65; 7.85]	5.2 [4.2; 8.1]	4.5 [4.3; 6.4]	0.195
Leukocytes (10^9^/l)	16.7 [8.1; 18.8]	7.8 [6.3; 21.2]	17.1 [13.5; 18.65]	16.7 [9.95; 28.7]	0.468
Platelets (10^9^/l)	183.0 [100.0; 252.0]	162.0 [55.0; 194.0]	187.0 [162.5; 284.0]	201.0 [77.0; 270.5]	0.310

Data presented as median [25th, 75th quantile]

*CK* creatin kinase*, CS* CytoSorb®, *CVVH c*ontinuous veno-venous hemofiltration*, CVVHD* continuous veno-venous hemodialysis*, HCO* high cut-off

According to the technical specification, the blood flow in CVVH was significantly higher to avoid hemoconcentration in the dialyzer compared with that of the two CVVHD—based procedures. The TTR was also higher with CVVH, but the difference was not significant (Additional file 4).

### Myoglobin clearance with CVVHD-HCO and CVVHD-CS compared to CVVH

The myoglobin elimination capacity was significantly higher after 12 h with CVVHD-HCO than with CVVH (*p* = 0.004). However, the differences in marginal means at the other time points were not significantly different.

**Fig. 2:**
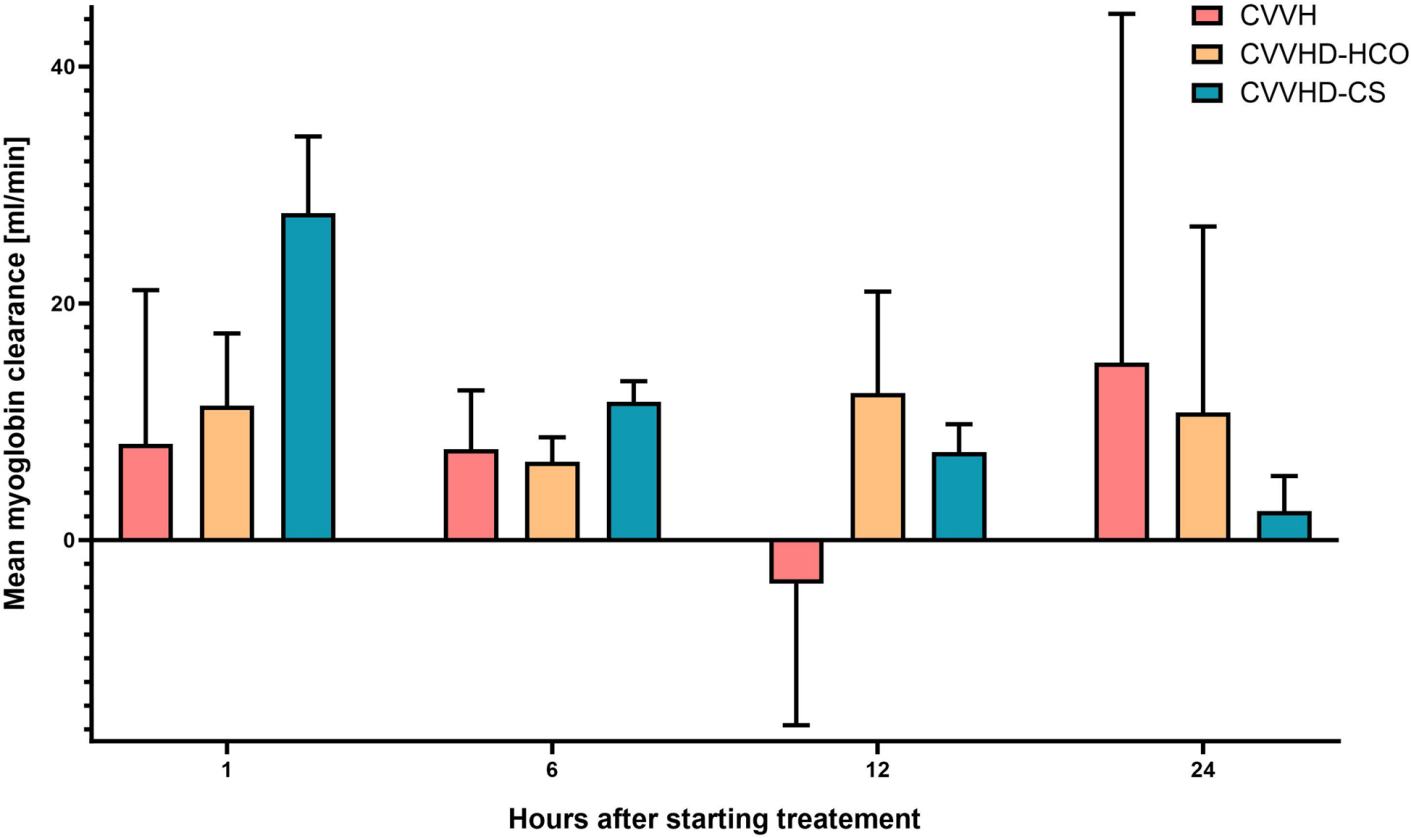
Mean myoglobin clearance Graphical data position as a bar chart with error bars. CI confidence interval, CVVH continuous veno-venous hemofiltration, CVVHD continuous veno-venous hemodialysis, HCO high cut-off

A significantly better myoglobin elimination was observed with CVVHD-CS after 1 h (*p* = 0.005) and 12 h (*p* = 0.019) compared to CVVH, while the difference in the marginal means was not significant after 6 h (*p* = 0.051). This difference became negative after 24 h (-12.57 (CI: -29.90–4.76)), indicating a decreasing elimination capacity with CVVHD-CS (Table 3, Fig. 2).

**Table 3:** Differences of marginal means of myoglobin clearances in CVVHD-HCO and CVVHD-CS (ml/min)^a^

**Time after starting KRT**	**n**	**CVVHD-HCO *n* = 5**	**CI**	***p***
1 h	15	3.39	-8.67–15.45	0.545
6 h	15	-0.96	-5.46–3.54	0.644
12 h	14	17.42	6.99–27.84	**0.004**
24 h	13	-4.24	-22.51–14.04	0.617
**Time after starting KRT**	**n**	**CVVHD-CS *n* = 5**	**CI**	***p***
1 h	15	18.37	6.923- 29.80	**0.005**
6 h	15	4.24	-0.03- 8,51	0.051
12 h	14	12.42	2.53- 22.31	**0.019**
24 h	13	-12.57	-29.90- 4.76	0.137

Data presented as marginal means and 95% confidence interval of marginal means

*CI* 95% confidence interval*, CS* CytoSorb®, *CVVH* continuous veno-venous hemofiltration*, CVVHD* continuous veno-venous hemodialysis*, HCO* high cut-off*, KRT* kidney replacement therapy

^a^The clearance in reference group (CVVH) was set equal to zero

### Substance specific clearances of other molecules

Elimination of small molecules was better with CVVH at the start of treatment (for urea at 1 h and 6 h; for creatinine at 1 h). The β2-microglobulin clearance was higher with CVVHD-CS one hour after initiation of the procedure (*p* = 0.007), although this kinetics was not maintained during further course. Similarly, the IL-6 clearance was higher after one (*p* = 0.015) and 6 h (*p* = 0.036) with CVVHD-CS, but also decreased over time. There was no relevant elimination of albumin in all investigated modes at all time points.

The calculated median clearance rates over the whole observation period did not significantly differ between the KRT modes for all investigated molecules (Table 4).

**Table 4:** Substance specific clearances (ml/min)

Time after starting KRT	n	CVVH *n* = 5	CVVHD-HCO *n* = 5	CVVHD-CS *n* = 5	*p*
**Myoglobin**					
1 h	15	4.82 [0.33; 17.61]	10.62 [7.78; 15.32]	28.16 [23.39; 31.57]	**0.014**
6 h	15	8.56 [4.53; 10.38]	6.11 [5.33; 8.16]	11.33 [10.56; 13.00]	**0.009**
12 h	14	-0.50 [-13.64; 4.72]	12.54 [7.30; 17.42]	6.93 [5.78; 9.31]	**0.031**
24 h	13	10.14 [0.53; 34.32]	6.00 [5.59; 20.70]	1.94 [0.38; 4.71]	0.089
**Cl**_**median**_	13	2.56 [-3.57; 15.25]	11.19 [8.32; 12.27]	9.60 [8.19; 10.35]	0.391
**Urea**					
1 h	15	40.00 [34.28; 47.81]	27.37 [22.67; 39.45]	25.19 [19.46; 30.25]	**0.031**
6 h	15	40.92 [35.67; 47.39]	25.83 [22.02; 37.15]	27.65 [20.47; 31.78]	**0.025**
12 h	14	40.00 [29.82; 46.28]	33.93 [23.86; 36.82]	30.72 [22.87; 36.82]	0.273
24 h	13	40.45 [34.73; 50.85]	25.36 [23.45; 41.06]	29.80 [25.13; 33.00]	0.096
**Cl**_**median**_	13	39.76 [32.11; 48.85]	32.12 [23.85; 37.92]	29.06 [22.78; 33.68]	0.136
**Creatinine**					
1 h	15	41.14 [35.06; 51.21]	31.42 [26.61; 41.72]	25.56 [20.98; 31.51]	**0.022**
6 h	15	43.01 [36.09; 48.85]	29.61 [28.00; 39.45]	28.25 [22.95; 35.66]	0.054
12 h	14	40.82 [29.27; 47.54]	36.48 [27.27; 40.20]	31.24 [25.26; 37.54]	0.343
24 h	13	39.97 [34.81; 52.02]	28.93 [27.62; 46.01]	31.43 [26.82; 36.16]	0.131
**Cl**_**median**_	13	41.12 [31.47; 50.11]	33.90 [28.05; 41.36]	30.52 [24.70; 35.75]	0.301
**β2-microglobulin**					
1 h	15	35.15 [25.12; 36.98]	26.41 [23.98; 33.73]	44.16 [41.89; 55.86]	**0.007**
6 h	15	28.71 [15.14; 33.07]	21.73 [18.87; 35.26]	30.83 [25.18; 32.94]	0.756
12 h	14	26.42 [2.72; 30.04]	21.83 [18.05; 32.00]	24.51 [22.97; 30.70]	0.741
24 h	13	32.39 [22.64; 45.80]	20.97 [19.14; 41.57]	18.98 [16.57; 22.02]	0.082
**Cl**_**median**_	13	28.94 [12.64; 31.29]	24.55 [19.08; 34.97]	27.40 [24.62; 30.63]	0.826
**Interleukin-6**					
1 h	15	10.09 [4.60; 18.45]	3.26 [2.07; 6.68]	19.18 [16.24; 22.70]	**0.015**
6 h	15	8.33 [4.65; 11.27]	1.71 [-0.61; 4.54]	8.06 [5.47; 9.88]	**0.036**
12 h	14	4.17 [2.21; 8.15]	1.55 [0.62; 2.38]	3.26 [3.01; 7.11]	0.056
24 h	13	10.84 [5.12; 34.38]	0.27 [-1.33; 16.71]	1.81 [0.14; 3.10]	0.067
**Cl**_**median**_	13	7.00 [5.57; 15.84]	1.53 [0.51; 6.30]	5.88 [5.68; 6.97]	0.205
**Albumin**					
1 h	15	1.34 [-13.23; 15.15]	-1.44 [-3.44; 0.93]	1.08 [0.14; 4.80]	0.310
6 h	15	0.86 [-3.14; 8.53]	-3.03 [-7.18; -1.35]	-2.08 [-3.44; 3.31]	0.230
12 h	14	-4.13 [-18.93; 3.95]	-2.60 [-7.18; 1.24]	-0.82 [-1.93; 0.07]	0.195
24 h	13	5.06 [-5.94; 32.31]	-4.28 [-5.11; 11.53]	0.32 [-1.27; 1.40]	0.446
**Cl**_**median**_	13	1.25 [-12.04; 6.35]	-2.05 [-6.12; 1.20]	-0.48 [-1.74; 1.49]	0.552

Data presented as median [25th, 75th quantile]

*Cl*_*median*_ calculated median clearance between 1 and 24 h, *CS* CytoSorb®*, CVVH* continuous veno-venous hemofiltration*, CVVHD* continuous veno-venous hemodialysis*, HCO* high cut-off*, KRT* kidney replacement therapy

### Time dependent elimination of molecules with CVVHD-CS

**Fig. 3:**
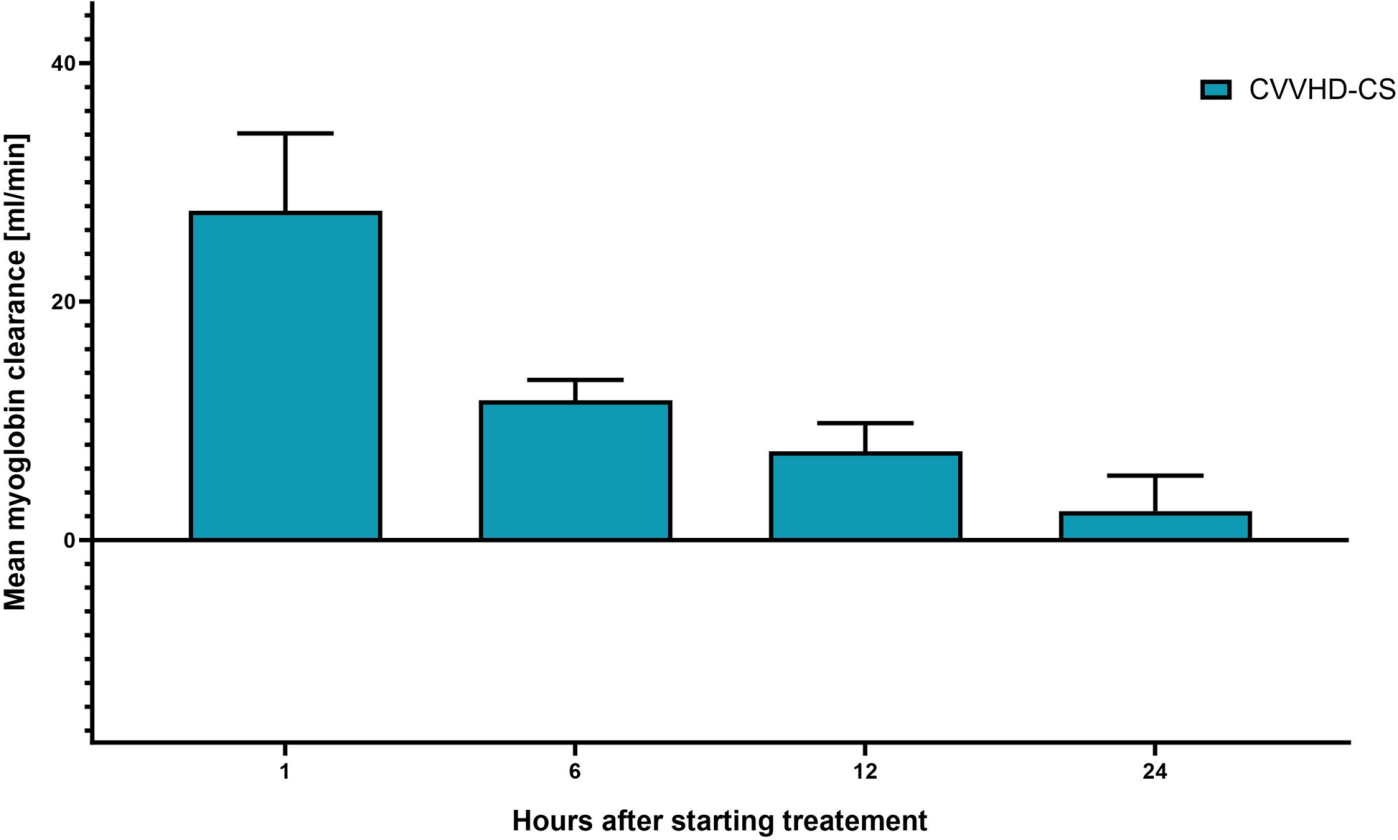
Myoglobin clearance in CVVHD-adsorber over time Graphical data position as a bar chart with error bars. *CI* confidence interval, *CVVHD* continuous veno-venous hemodialysis

While myoglobin elimination using CVVHD-CS was the highest at the beginning, this clearance sharply decreased over time (Fig. 3). This effect was significant using a general linear model for this group (*p* = 0.023), while this could not be proved for the other modes (CVVH: *p* = 0.125, CVVHD-HCO: *p* = 0.644). This observation was confirmed by the decreasing substance-specific clearances of other molecules with middle molecule weight such as β2-microglobulin (*p* < 0.001) and IL-6 (*p* = 0.029). In contrast, the elimination performance for small molecules was stable during the observation period (urea (*p* = 0.206) and creatinine (*p* = 0.155)). There were no significant changes regarding albumin clearance during the study time points (*p* = 0.090) (Table 5).

**Table 5:** Substance-specific clearances in CVVHD-CS over time

Parameter, time after starting KRT	n	mean	CI	P^*^
**Myoglobin**				
1 h	5	27.62	21.13–34.10	
6 h	5	11.69	9.96–13.42	**0.002**
12 h	5	7.42	5.07–9.78	**0.001**
24 h	5	2.43	-0.56–5.41	** < 0.001**
**Urea**				
1 h	5	24.92	17,63–32.21	
6 h	5	26.43	17.68–35.19	0.496
12 h	5	30.02	20.33–39.72	0.077
24 h	5	29.21	22.99–35.44	0.098
**Creatinine**				
1 h	5	26.11	19.34–32.87	
6 h	5	29.09	19.72–38.47	0.365
12 h	5	31.37	22.36–40.37	0.135
24 h	5	31.48	24.33–38.64	0.090
**β2-microglobulin**				
1 h	5	47,9333	38.78–57.08	
6 h	5	29,4148	24.18–34.65	**0.005**
12 h	5	26,3672	20.05–32.69	**0.008**
24 h	5	19,2308	15.73–22.73	**0.001**
**Interleukin-6**				
1 h	5	19.41	14.63–24.20	
6 h	5	7.75	4.83–10.68	**0.006**
12 h	5	4.70	1.95–7.46	**0.002**
24 h	5	1.66	-0.31–3.62	**0.001**
**Albumin**				
1 h	5	2.19	-1.65–6.03	
6 h	5	-0.47	-5.71–4.78	**0.043**
12 h	5	-0.91	-2.37–0.56	**0.048**
24 h	5	0.12	-1.78–2.02	0.118

Data presented as means and 95% confidence interval of means

*CI* 95% confidence interval*, CS* CytoSorb®*, CVVHD* continuous veno-venous hemodialysis*, KRT* kidney replacement therapy

^*^general linear model with repeated measurement (reference category: 1 h value)

### Specific clearance performance of the adsorber and the high-flux dialyzer with CVVHD-CS

The calculated Cl_int_ for the middle molecular weight substances such as myoglobin and IL-6 immediately after the adsorber was higher compared to the final Cl_post_ after 6, 12 and 24 h. In contrast, the Cl_int_ of urea and creatinine was nearly zero. These substances were only removed through the high-flux dialyzer. For β2-microglobulin, hemoadsorption and hemodialysis have an additive effect with an increasing clearance along the extracorporeal circuit. While the elimination of albumin in the CVVHD-CS group was negligible, the calculated clearance rate for albumin was higher at Cl_int_ after 24 h (Table 6).

**Table 6:** Substance specific clearances between (Cl_int_) and after (Cl_post_) CS and dialyzer (ml/min)

Time after starting KRT	n	Cl_int_	Cl_post_	P^*^
**Myoglobin**				
1 h	5	29.33 [24.20; 32.47]	28.16 [23.39; 31.57]	0.138
6 h	5	14.37 [12.79; 15.75]	11.33 [10.56; 13.00]	**0.043**
12 h	5	10.52 [9.27; 11.54]	6.93 [5.78; 9.31]	**0.043**
24 h	5	5.30 [4.22; 6.56]	1.94 [0.38; 4.71]	**0.043**
**Urea**				
1 h	5	1.77 [0.97; 2.48]	25.19 [19.46; 30.25]	**0.043**
6 h	5	1.51 [0.99; 2.71]	27.65 [20.47; 31.78]	**0.043**
12 h	5	4.05 [0.72; 4.49]	30.72 [22.87; 36.82]	**0.043**
24 h	5	0.76 [-0.43; 3.56]	29.80 [25.13; 33.00]	**0.043**
**Creatinine**				
1 h	5	0.56 [-0.38; 1.18]	25.56 [20.98; 31.51]	**0.043**
6 h	5	2.39 [1.41; 3.09]	28.25 [22.95; 35.66]	**0.043**
12 h	5	2.17 [1.63; 3.12]	31.24 [25.26; 37.54]	**0.043**
24 h	5	1.57 [0.00; 3.80]	31.43 [26.82; 36.16]	**0.043**
**β2-microglobulin**				
1 h	5	37.87 [36.77; 46.86]	44.16 [41.89; 55.86]	**0.043**
6 h	5	19.10 [17.24; 23.10]	30.83 [25.18; 32.94]	**0.043**
12 h	5	14.09 [12.56; 20.07]	24.51 [22.97; 30.70]	**0.043**
24 h	5	9.67 [7.41; 10.43]	18.98 [16.57; 22.02]	**0.043**
**Interleukin-6**				
1 h	5	21.86 [17.40; 23.95]	19.18 [16.24; 22.70]	0.068
6 h	5	11.50 [8.57; 13.63]	8.06 [5.47; 9.88]	**0.043**
12 h	5	6.18 [5.96; 8.81]	3.26 [3.01; 7.11]	**0.043**
24 h	5	4.83 [3.54; 6.10]	1.81 [0.14; 3.10]	**0.043**
**Albumin**				
1 h	5	4.45 [1.07; 5.51]	1.08 [0.14; 4.80]	0.500
6 h	5	0.91 [-1.24; 2.81]	-2.08 [-3.44; 3.31]	0.500
12 h	5	0.89 [-3.22; 2.67]	-0.82 [-1.93; 0.07]	0.500
24 h	5	3.26 [1.23; 4.60]	0.32 [-1.27; 1.40]	**0.043**

Data presented as median [25th, 75th quantile]

*CS* CytoSorb®*, KRT* renal replacement therapy

^*^Wilcoxon signed-rank test

### Safety endpoints and lifetime of extracorporeal circuit

Adverse events and severe adverse events did not differ significantly between the groups. Two patients with CVVHD-HCO died during the intervention due to a refractory septic shock. Bleeding complications and clotting of extracorporeal circuit were observed only in the CVVH group (Table 7). There was no difference regarding the ICU length of stay between the groups. A total of 11 patients died, with no difference between the groups regarding overall mortality rates. The cause of death was sepsis in six patients, acute heart failure in three, hypoxic ischemic encephalopathy and acute on chronic liver failure in one each.

**Table 7:** Safety endpoints

Parameter	All *n* = 15	CVVH *n* = 5	CVVHD-HCO *n* = 5	CVVHD-CS *n* = 5	*p*
ICU stay (d)	10 [4; 31]	12 [5; 51]	10 [1; 29]	8 [6; 21]	0.689
ICU mortality n, (%)	9/15 (60)	3/5 (60)	3/5 (60)	3/5 (60)	1.000
hospital mortality n, (%)	11/15 (73)	4/5 (80)	4/5 (80)	3/5 (60)	0.711
28d mortality n, (%)	8/15 (53)	2/5 (40)	3/5 (60)	3/5 (60)	0.765
90d mortality n, (%)	11/15 (73)	4/5 (80)	4/5 (80)	3/5 (60)	0.711
**SAE**					
Treatment associated life-threatening complications n, (%)	0/15 (0)				
Dead of any cause during intervention n, (%)	2/15 (13)	0/5 (0)	2/5 (40)^a^	0/5 (0)	0.099
**AE**					
Hypocalcaemia n, (%)	1/15 (7)	0/5 (0)	0/5 (0)	1/5 (20)	0.343
Metabolic alkalosis n, (%)	0/15 (0)				
Citrate accumulation n, (%)	1/15 (7)	0/5 (0)	1/5 (20)	0/5 (0)	0.343
Bleeding with transfusion n, (%)	0/15 (0)				
Bleeding without transfusion n, (%)	2/15 (13)	2/5 (40)	0/5 (0)	0/5 (0)	0.099
Clotting of extracorporeal circuit n, (%)	2/15 (13)	2/5 (40)^b^	0/5 (0)	0/5 (0)	0.099
HIT n, (%)	0/15 (0)				
Dysfunction of dialysis catheter n, (%)	2/15 (13)	1/5 (20)	1/5 (20)	0/5 (0)	0.562

Data presented as *n* (%) or median [25th, 75th quantile]

*AE* adverse event*, CS* CytoSorb®*, CVVH* continuous veno-venous hemofiltration*, CVVHD* continuous veno-venous hemodialysis*, HCO* high cut-off*, HIT* heparin-induced thrombocytopenia*, ICU* intensive care unit*, SAE* sever adverse event

^a^dead 10 h and 29 h after initiation CVVHD-HCO

^b^clotting of extracorporeal circuit 14 h and 25 h after initiation of CVVH

The median filter lifespans for the different KRT modes were 60 h [0; 135] in CVVH, 61 h [0; 132] in CVVHD-HCO and 67 h [42; 92] in CVVHD-CS group. These differences were not significant (*p* = 0.863).

### Myoglobin clearance with simulated alternative KRT settings

The potential performance of alternative KRT settings were estimated. First, a model combining CVVHD-HCO with the adsorber gave a median myoglobin clearance of 20.79 ml/min. Second, a more frequent adsorber exchange (every 12 h) in a CVVHD with a standard high-flux dialyzer resulted in a median myoglobin clearance of 14.91 ml/min. Finally, both tested models were merged. CVVHD-HCO combined with the adsorber with a 12-hourly adsorber exchange could presumably generate a myoglobin clearance of 26.10 ml/min.

## Discussion

In this prospective randomized controlled pilot trial on critically ill patients with RM and severe AKI requiring KRT, myoglobin elimination using CVVHD with a high-flux dialyzer in combination with an adsorber was found to be the most effective mode during the first hours of treatment. This finding is supported by other studies that also showed a high elimination capacity for myoglobin in the early stages of treatment by combining CVVHD with CS [16–18]. The high myoglobin elimination rates within the first hour reported by those studies suggests that our findings on the performance of the CVVHD combined with the adsorber may have been underestimated, because we did not take into account the effects of the first treatment hour.

Nevertheless, there were no significant differences between the three studied modes regarding the average myoglobin clearance during the first 24 h of treatment. This can be explained by the rapid decline in myoglobin clearance in CVVHD-CS during the longer observation period. A retrospective comparative study in patients with severe RM, notably with a short treatment period of only 6–12 h, has also shown no differences in the myoglobin reduction rate between intermittent hemodiafiltration with high cut-off dialyzer, intermittent hemodialysis with medium cut-off dialyzer and CVVHD-CS [15].

CVVH has been defined as the control mode based on previous reports that proposed this as the standard procedure for myoglobin elimination in AKI [2, 4]. However, despite similar TTRs, we have observed considerably lower myoglobin clearance rates with this mode compared to that reported by Amyot using a 0.9m^2^ polyacrylnitril (AN69) dialyzer [11]. The myoglobin clearance using CVVHD-HCO was similar to that in our previous study [14].

Small molecules such as urea and creatinine were best eliminated with CVVH, most probably due to the significantly higher blood flow and TTR [29]. The higher blood flow is required in order to avoid a critical hemoconcentration along the dialyzer in the postdilution mode [30].

Similar to the elimination kinetics for myoglobin, the best initial clearance for the other middle molecular weight substances β2-microglobulin and IL-6 was found in CVVHD-CS. In due course and when averaged over time, no differences in clearance could be observed between the various modes. The high IL-6 clearance, a pro-inflammatory cytokine, might be an interesting adjunctive therapeutic approach for patients with sepsis. Although case series and retrospective studies on patients with septic shock demonstrated improved survival using hemoperfusion with CS [31, 32], no survival benefit has so far been documented in prospective studies [33].

β2-microglobulin, on the other hand, is a surrogate parameter for medium molecular weight uremic toxins. However, the clinical effects of reducing serum concentrations through extracorporeal measures are still uncertain [13, 34].

Finally we did not observe any relevant loss of albumin with the three modes studied. This was not surprising since the cutoff for the dialyzers applied is 30 kDa. Previous elimination studies using CVVHD-HCO with the EMiC2 filter also showed no albumin loss [13, 14]. There is currently a lack of clinical data regarding a potential albumin adsorption with the Cytosorb adsorber. Since the cut-off for this adsorber with 55 kDa [17] is not far lower than the molecular mass of albumin (66.5 kDa), minimal albumin loss could be assumed.

Increasing saturation of the adsorber during CVVHD-CS leads to a loss of elimination ability despite the extensive adsorption surface of 45,000 m^2^ [17]. In contrast, the elimination performance of a dialyzer is largely maintained by continuous fresh dialysis or substitution solution and is only slightly affected by membrane fouling over time [35, 36]. Other recently published reports have also shown a rapid decline in myoglobin and IL-6 elimination performance using the combination of continuous KRT and CS [16, 18, 37]. This study showed that myoglobin (17.1 kDa) and IL-6 (26.0 kDa) are only removed with the adsorber in CVVHD-CS. For the smaller β2-microglobulin (11.8 kDa), elimination obviously occurs through both the adsorption and the dialysis. Over time, the elimination decreased until the adsorber was saturated, while the elimination with dialysis remained almost constant. Surprisingly, Cl_int_ was slightly higher than Cl_post_ for myoglobin and IL-6. Filtration-backfiltration processes may explain this phenomenon [38]. Despite the negligible clearance, this phenomenon could also be observed for albumin. Creatinine and urea were hardly removed by the adsorber in this study. Although the adsorber binds a wide variety of hydrophobic substances up to a molecular weight of 55 kDa [17, 39], it does not seem to play a role in the elimination of small hydrophilic molecules. These are almost completely eliminated by the dialysis part of the system [16].

We did not observe any significant differences between the groups regarding ICU-length of stay, hospital-, 28- and 90-day mortality. However, these findings should be carefully interpreted due to the small sample sizes. The very high 90-day mortality of 73% is in the range of other studies on critically ill patients with AKI, RM and need for KRT [8, 14, 40]. The SOFA score and the need for KRT were recently identified as independent risk factors for ICU mortality in patients with RM [8].

In the group with CVVH, two patients experienced bleeding problems and the extracorporeal circuit was clotted before the scheduled end of treatment in two patients. This observation is in line with the findings of a recent multicenter study [41]. To avoid these problems of systemic anticoagulation, performing CVVH with RCA could be a possible alternative. However, due to the required higher blood flow, there would be a significant increase in citrate intake with the risk of citrate accumulation. A high disease severity, such as observed in this study, is often associated with citrate accumulation [42].

Using the calculated clearance performances of the different KRT modes, our study showed the modelling of alternative KRT settings with the aim of further increasing myoglobin elimination. The knowledge gained from the additional determination of Cl_int_ in CVVHD-CS was a prerequisite for the corresponding calculations. Higher effectiveness was recently demonstrated for the combination of CVVHD-HCO and CS [16]. Additionally, based on the observed saturation kinetics of the adsorber, a model with an adsorber exchange every 12 h would increase the median myoglobin clearance. A recently published consensus paper also recommends an adsorber exchange every 12 h until the serum myoglobin concentration falls below 10,000 ug/l [19]. Finally, the model combination of CVVHD-HCO and CS with a 12-hourly adsorber exchange resulted in a sustained high median myoglobin clearance throughout the entire assumed treatment period. The effectiveness of this concept has been demonstrated in a case report [43].

### Strengths

In this study, various options for myoglobin elimination were investigated for the first time in a prospective randomized design in critically ill patients with RM and AKI requiring KRT. Our trial provides precise data on the performance of the different modes studied and may help in decision making regarding the appropriate procedure.

### Limitations

Our study has several limitations. Firstly, because of the small sample size, it is possible that relevant differences may have been missed (type 2 error). Secondly, there is a risk of a type 1 error due to multiple testing. Thirdly, the substance-specific elimination performance in CVVH may have been overestimated due to the higher blood flow and TTR. Fourth, the small sample size does not allow any valid statement on clinical outcome. Fifth, we did not take into account the effects of the first treatment hour. Thus, the high myoglobin elimination rate in CVVHD-CS within the first hour reported by previous studies [16, 18] suggests that the performance of the CVVHD-CS may have been underestimated. Finally, possible differences in baseline myoglobin concentration may have affected clearance performance over time due to the saturation kinetics of the adsorber. Nevertheless, this study allows the characterization of the elimination capacity of the examined KRT modes over time in RM.

## Conclusions

CVVHD-CS provides by far the highest myoglobin clearance in the first hours of treatment. However, due to the rapid decline of the elimination capacity over the period of 24 h, no difference in the averaged clearance over time could be observed between CVVHD-CS, CVVHD-HCO and CVVH. In CVVH with heparin-based anticoagulation, the risk of bleeding or clotting was more frequent. Therefore, the old standard CVVH for the treatment of AKI in RM must be critically questioned in view of the newer available alternatives.

Theoretically, a further increase in myoglobin clearance could be achieved with the combination of CVVHD-CS-HCO with a 12-hourly exchange of the adsorber. However, large clinical trials are necessary to evaluate whether faster elimination of myoglobin could influence clinical outcome parameters.

## Supplementary Information

Supplementary Material 1.

Supplementary Material 2.

Supplementary Material 3.

Supplementary Material 4.

Supplementary Material 5.

## Data Availability

The datasets analyzed during the current study are available from the corresponding author on reasonable request.

## Abbreviations

AE: Adverse events
AKI: Acute kidney injury
APACHE: II Acute Physiology And Chronic Health Evaluation II
aPTT: Activated partial thromboplastin time
C_post_: Substance specific concentration after the dialyzer
C_pre_: Substance specific concentration before the dialyzer
CKD: Chronic kidney disease
Cl_1h, 6__h__, 12__h__, 24__h_: Plasma clearance at different time points
Cl_corr_: Corrected plasma clearance
Cl_int_: Plasma clearance between adsorber and dialyzer
Cl_median_: Median plasma clearance
Cl_p_: Substance-specific plasma clearance
Cl_post_: Plasma clearance after adsorber and dialyzer
C_post,corr_: Corrected plasma concentration
Cl_total_: Total plasma clearance
CS: CytoSorb®
CVVH: Continuous veno-venous hemofiltration
CVVHD: Continuous veno-venous hemodialysis
CVVHD-CS: Continuous veno-venous hemodialysis with the additional adsorber CytoSorb®
CVVHD-HCO: Continuous veno-venous hemodialysis using a high cut-off dialyzer
ECLIA: Electrochemiluminescence immunoassay
FP: Filtration portion
HCO: High cut-off
hct: Hematocrit level
ICU: Intensive care unit
IL-6: Interleukin-6
KRT: Kidney replacement therapy
Qb: Blood flow
Qp_pre_: Plasma flow in the extracorporeal circuit
Qp_post_: Postfilter plasma flow
RCA: Regional citrate anticoagulation
RM: Rhabdomyolysis
SAE: Severe adverse events
SOFA: Sequential Organ Failure Assessment
TTR: Total turnover rate
