# Supplementary material for: Kidney REPLACEment therapies in patients with acute kidney injury and RHABDOmyolysis (ReplaceRhabdo): a pilot trial
Source: BMC Nephrol. 2025 Jan 14;26:23. doi: 10.1186/s12882-025-03945-3 (PMC11731544; doi:10.1186/s12882-025-03945-3)
Supplement: Supplementary file 2 — Supplementary Material 2. [file 12882_2025_3945_MOESM2_ESM.docx]

**Additional file 2: Calculations**

Plasma flow in the extracorporeal circuit (*Qp_pre_*) was calculated using blood flow (*Qb*) of extracorporeal circuit and patient`s hematocrit level (*hct*) at the time of sampling:

$Qp$_pre_$(ml/min)=Qb\times((1-hct)\div100)$

The substance-specific plasma clearance (*Cl_p_*) was calculated with the substance specific concentration before (C_pre_) and after (C_post_) the dialyzer:

$Clp (ml/min) = Qp$*_pre_*$\times((C$*_pre_*$- C$*_post_*$) \div C$*_pre_*$)$

The respective total elimination between 1h and 24h (*Cl_total_ (ml/23h))* was determined using following formula and the median clearances:

$${Cl}_{total}\left( ml/23h \right)=(({Cl}_{1h}+{Cl}_{6h})\div2)\times5\times60+(({Cl}_{6h}+{Cl}_{12h})\div2)\times6\times60+(({Cl}_{12h}+{Cl}_{24h})\div2)\times12\times60$$

Between initiation of KRT and 1h, the elimination capacity at this period of equilibration is not known and therefore consequently not included into the calculation of median plasma clearance (Cl_median_):

$${Cl}_{median}(ml/min)={Cl}_{total}\div(23\times60)$$

As a sampling port is only available before the influx of the replacement fluid into the extracorporeal circuit in CVVH, the postfilter solute concentration (C_post_) had to be corrected to account for the replacement fluid flow. Following this, we calculated postfilter plasma flow (*Qp_post_*) by subtracting filtration portion (*FP*, ml/min) from the pre-filter plasma flow (*Qp_pre_*):

$${Qp}_{post}(ml/min)={Qp}_{pre}-FP$$

The corrected plasma concentration was calculated using the following formula:

$$C_{post,corr}(mmol/l)=({Qp}_{post}\div{Qp}_{pre})\times C_{post}$$

Finally, the corrected plasma clearance (*Cl_corr_*) could then be calculated using the following formula:

$${Cl}_{corr}(ml/min)={Qp}_{pre}\times((C_{pre} -C_{post,corr})\div C_{pre})$$

The developed clearance formulas were then applied to create models with a 12 hourly exchange of the adsorber and to test the combination of different KRT procedures. The clearance power for an adsorber change every 12 hours was calculated according to the following formulas:

$${Cl}_{total, 12h}\left( ml/11h \right)=(({Cl}_{1h}+{Cl}_{6h})\div2)\times5\times60+(({Cl}_{6h}+{Cl}_{12h})\div2)\times6\times60$$

$${Cl}_{median, 12h}(ml/min)={Cl}_{total,12h}\div(11\times60)$$

In order to calculate the combinations of different KRT procedures, the clearance values have been added together.
