# Supplementary material for: Kidney REPLACEment therapies in patients with acute kidney injury and RHABDOmyolysis (ReplaceRhabdo): a pilot trial
Source: BMC Nephrol. 2025 Jan 14;26:23. doi: 10.1186/s12882-025-03945-3 (PMC11731544; doi:10.1186/s12882-025-03945-3)
Supplement: Supplementary file 3 — Supplementary Material 3. [file 12882_2025_3945_MOESM3_ESM.docx]

**Additional file 3: Laboratory analyses**

- Urea: kinetic test with urease and glutamate dehydrogenase, module c701
- Creatinine: enzymatic method with creatinase, module c701
- ß2-microglobulin: turbidimetry, module c701
- Myoglobin: electrochemiluminescence immunoassay (ECLIA), module e801
- IL-6: electrochemiluminescence immunoassay (ECLIA), module e801
- Human albumin: color test with bromocresol green, module c701
