# Supplementary material for: Kidney REPLACEment therapies in patients with acute kidney injury and RHABDOmyolysis (ReplaceRhabdo): a pilot trial
Source: BMC Nephrol. 2025 Jan 14;26:23. doi: 10.1186/s12882-025-03945-3 (PMC11731544; doi:10.1186/s12882-025-03945-3)
Supplement: Supplementary file 4 — Supplementary Material 4. [file 12882_2025_3945_MOESM4_ESM.docx]

**Additional file 4: Protocol of kidney replacement therapy**

| **Time after starting KRT** | **n** | **CVVH n=5** | **CVVHD-HCO n=5** | **CVVHD-CS n=5** | ***p*** |
| --- | --- | --- | --- | --- | --- |
| **Blood flow (ml/min)** |  |  |  |  |  |
| 1h | 15 | 210 [190; 240] | 100 [95; 125] | 100 [80; 105] | **0.006** |
| 6h | 15 | 210 [190; 240] | 120 [95; 125] | 100 [80; 115] | **0.006** |
| 12h | 14 | 210 [190; 240] | 110 [93; 128] | 100 [80; 120] | **0.010** |
| 24h | 13 | 215 [185; 238] | 115 [93; 138] | 100 [80; 120] | **0.017** |
| **Total turnover rate (ml/h)** |  |  |  |  |  |
| 1h | 15 | 2400 [2050; 2790] | 2000 [1800; 2500] | 2000 [1550; 2025] | 0.089 |
| 6h | 15 | 2400 [2050; 2790] | 2000 [1800; 2500] | 2000 [1550; 2025] | 0.089 |
| 12h | 14 | 2400 [2050; 2790] | 2200 [1750; 2500] | 2000 [1550; 2275] | 0.237 |
| 24h | 13 | 2375 [2025; 2860] | 2200 [1750; 2500] | 2000 [1550; 2275] | 0.237 |

Data presented as median [25th, 75th quantile]

*CS* CytoSorb®*, CVVH* continuous veno-venous hemofiltration*, CVVHD* continuous veno-venous hemodialysis*, HCO* high cut-off*, KRT* renal replacement therapy
