# Supplementary material for: Kidney REPLACEment therapies in patients with acute kidney injury and RHABDOmyolysis (ReplaceRhabdo): a pilot trial
Source: BMC Nephrol. 2025 Jan 14;26:23. doi: 10.1186/s12882-025-03945-3 (PMC11731544; doi:10.1186/s12882-025-03945-3)
Supplement: Supplementary file 5 — Supplementary Material 5. [file 12882_2025_3945_MOESM5_ESM.docx]

**1.Titel**

Different renal REPLACEment therapies in patients with acute kidney injury and RHABDOmyolysis (ReplaceRhabdo): A pilot trial

**2. Abstract**

| **Titel** | Different renal REPLACEment therapies in patients with acute kidney injury and RHABDOmyolysis (ReplaceRhabdo): A pilot trial |
| --- | --- |
| **Investigator** | Lorenz Weidhase MD (PI) Jonathan de Fallois (Co-PI) Sirak Petros MD |
| **Primary endpoint** | - Difference of marginal means in myoglobin clearance |
| **Secondary endpoints** | - Clearance of other molecules with different molecule weight (creatinine, urea, beta-2 microglobulin, Il-6, albumin) after 1h, 6h, 12h and 24 hours - Lifetime of extracorporeal circuit (filter lifetime) - 90 day outcome: Composite endpoint of patient dead, persistent loss of renal function requiring dialysis or persistent decline in GFR (>25% of baseline value) - Serum myoglobin before and after RRT - Clinical endpoints:   - ICU length of stay (days)   - ICU mortality   - Hospital mortality   - Mortality day 28   - Mortality day 90 - Safety endpoints (Intervention period 72 hours):   - Adverse events:     - Hypocalcemia     - Metabolic alkalosis     - Citrate accumulation     - Bleeding requiring blood transfusion     - Heparin induced Thrombocytopenia (HIT)     - Catheter malfunction   - Severe adverse events:     - Treatment-associated life-threatening complication     - Dead of any cause during intervention |
| **Inclusion criteria** | - informed consent - Acute kidney injury requiring RRT (AKIN III) - rhabdomyolysis with myoglobinemia >4000 µg/l |
| **Exclusion criteria** | - refuse to participate - refusal of RRT - age < 18 years - contraindication for RRT (e.g. end-stage malignant tumor) - Preexisting end stage kidney disease (CKD G5)/chronic intermittent hemodialysis - Heparin-induced thrombozytopenia - Pregnancy and lactation - Participation in other treatment studies |
| **Procedure** | - informed consent - randomization 1:1:1 arm A, B or C (CVVH, CVVHD-HCO, CVVHD-Adsorber) - blood test pre-, post- and in group C additional int-dialyzer after 1h, 6h, 12h and 24h as well as calculation of substance specific clearances - follow-up after intervention (day 90):   - dialyzer lifetime   - renal and clinical outcome |
| **Approximated recruitment period** | 01.03.2021-28.02.2022 |
| **Study center** | University Hospital Leipzig, medical ICU |
| **Funding** | CytoSorbents Europe GmbH, Müggelseedamm 131, 12587 Berlin |
| **Patient calculation** | Patients will be screened: n = 36 Patients will be included: n = 15 |

**3. Investigators**

Lorenz Weidhase MD (PI)

Medical ICU

University hospital Leipzig

Liebigstraße 20

04103 Leipzig

Jonathan de Fallois (Co-PI)
Devision of nephrology, medical clinic III
University hospital Leipzig
Liebigstraße 20
04103 Leipzig

Prof. Sirak Petros MD

Medical ICU

University hospital Leipzig

Liebigstraße 20

04103 Leipzig

**4. Funding**

CytoSorbents Europe GmbH, Müggelseedamm 131, 12587 Berlin

**5. Registry**

German trial registry (DRKS) *and* approved by the local ethics committee (ethical committee at the medical faculty, Leipzig university, 361/20-ek).

**6. Background**

Acute kidney injury (AKI) is one of the leading organ dysfunctions in critically ill patients and is associated with high mortality and morbidity rates, particularly if renal replacement therapy (RRT) is required.^1^ In most cases several pathogenetic mechanisms are involved in the development of AKI in critically ill patients.^2^ Rhabdomyolysis is considered as the potential cause of approximate 10% of acute kidney injuries.^3^ Damaged skeletal muscle releases muscle cell contents into the circulation, e.g., myoglobin and other proteins.
Myoglobin is endocytosed by tubular cells and oxidized, resulting in radical oxygen species that alter DNA and proteins. It activates an inflammatory response in the kidney and mediates vasoconstriction, which perpetuates renal damage. Myoglobin is also filtered by the glomerulus and precipitates in the renal tubules, particularly in combination with the Tamm–Horsfall proteins, forming tubular casts, consequently result in acute tubular obstruction.^3,4^

There is no uniform definition of rhabdomyolysis. Most studies define muscle cell damage on the basis of elevated creatinine kinase (CK).^5^ Recent data imply that myoglobin might be a better parameter, because it predicts development of AKI in these cases.^6^ A peak value of myoglobin >3865 µg/l is considered to be a good predictor for AKI.^7^

Fast elimination of myoglobin seems essential due to its direct toxic renal effects,^4^ however clinical data are limited referring efficacy of RRT in rhabdomyolysis. Diffusion based hemodialysis is usually not able to eliminate uremic molecules with middle molecule weight, for example myoglobin (17 kDa). Elimination of them is feasible by convective transport (hemofiltration). On this account application of continuous veno-venous hemofiltration (CVVH) is recommended in patients with AKI and rhabdomyolysis.^8,9^ A Cochrane analysis concerning this issue reviews only three Chinese studies with in total 101 patients. It compares continuous renal replacement therapy (CRRT) by CVVH with conventional therapy by intermittent hemodialysis as appropriate. In this study CRRT reduced myoglobin and creatinine levels faster and led to prior recovery of diuresis.^10^

Hemofiltration requires higher extracorporeal circuit blood flow than hemodialysis, which is related to hemoconcentration at the dialyzer. Therefore, regional citrate anticoagulation (RCA) during CVVH is hardly possible due to the high risk of citrate accumulation. Usually, CVVH requires systemic anticoagulation with heparin. Systemic anticoagulation is associated with more bleeding complications, shorter lifespan of the dialyzer and higher incidence of heparin-induced thrombocytopenia (HIT) than RCA.^11^ Current international guidelines recommend anticoagulation with citrate for CRRT, unless systemic anticoagulation is required for other indications or citrate use is contraindicated.^12^

There are some opportunities to avoid systemic anticoagulation and maintain middle molecule clearance and thus maintain myoglobin elimination. One opportunity could be the application of high cutoff (HCO) membranes with a pore size larger than 0.01 μm in CVVHD (CVVHD-HCO).^13–15^ Application of an additional extracorporeal blood adsorber in CVVHD could be another opportunity to eliminate myoglobin. In recent years blood adsorber were an approach to treat cytokine release syndromes such as septic shock.^16,17^ These adsorbers bind not only cytokines (for example Interleukin 6, Il-6), but also myoglobin and elimination of it seems possible.^18^

This pilot trial is supposed to evaluate the individual therapeutic benefit for critical care patients by application of HCO membranes or adsorber in CVVHD using RCA compared to CVVH with systemic anticoagulation. Advantage of both methods is the minimization for the risk of bleeding and HIT. Probably the methods are superior in the elimination of toxic myoglobin compared to CVVH.
Most recently we demonstrated that the application of CVVHD-HCO has got a higher myoglobin clearance than the application of continuous veno-venous hemodiafiltration (CVVHDF).^15^ Case reports indicate that blood adsorber might eliminate myoglobin even more effective.^19^ Application of blood adsorbers is official approved for rhabdomyolysis.

The three above mentioned opportunities of RRT in rhabdomyolysis were regular used during the last years at the medical ICU at the university hospital Leipzig. There are no reliable findings at present, which renal replacement therapy is the best choice in this situation.

In detail, we plan to enroll patients with acute kidney injury requiring RRT and suffering from rhabdomyolysis. Eligible patients will be randomized in the three therapy groups. Myoglobin clearance is calculated as surrogate parameter and the clearance of other molecules with different molecule weight. Renal outcome will be monitored.

In our opinion CVVHD with high cutoff filter or adsorber using RCA is at least effective then CVVH using systemic anticoagulation in the treatment of AKI and rhabdomyolysis and reduces the risk of complications. On this account it might improve renal outcome.

The trial is a pilot trial. The objectives of the study are: Firstly, to evaluate different renal replacement therapy in the context of severe rhabdomyolysis and secondly, to test and generate potential clinical outcome parameters for further prospective studies with a larger number of participants.

**7. Methods**

**7.1. Study objectives and design:**

We plan a prospective, randomized, single-blinded, single-center pilot trial. Informed consent is necessity and either given by the patients or their legal guardians. Allocation concealment and unrestricted randomization will carried out using sequentially numbered, opaque sealed envelopes as previously described.^20^ For technical reasons, only patients can be blinded to the treatment arm.

**7.2.1. Inclusion criteria:**

- Informed consent
- Acute kidney injury requiring renal replacement therapy (according KDIGO guidelines)^12^
- Plasma myoglobin level above 4000 µg/l.

**7.2.2. Exclusion criteria:**

- Refuse to participate
- Rejection of renal replacement therapy
- Age < 18 years
- Contraindication for renal replacement therapy, for example end-stage underlying disease
- HIT
- Preexisting end stage kidney disease (CKD G5)/chronic intermittent hemodialysis
- Pregnancy and lactation
- Participation in other treatment studies

**7.3. Patients:**

All patients who are admitted to our medical ICU will be screened for eligibility during the recruitment period. If they meet inclusion criteria patients will be randomized 1:1:1 to one of the following treatment groups:

Group A CVVH-postfilter with high-flux filter (Ultraflux® AV1000S, Fresenius Medical Care AG & Co., Else-Kroener-Straße 1, Bad Homburg, Germany)

Group B CVVHD with HCO filter (EMiC®2, Fresenius Medical Care AG & Co., Else-Kroener-Straße 1, Bad Homburg, Germany)

Group C CVVHD with high-flux filter (Ultraflux® AV1000S, Fresenius Medical Care AG & Co., Else-Kroener-Strasse 1, Bad Homburg, Germany) and adsorber (CytoSorb®, CytoSorbents Europe GmbH, Müggelseedamm 131, Berlin, Germany)

**7.4. Procedure:**

A central venous access using a 13 French double-lumen high-flow catheter (Achim Schulz-Lauterbach VMP, Iserlohn, Germany) will be placed. The control group A will be managed with CVVH postdilution using the high-flux dialyzer Ultraflux AV1000S (Fresenius Medical Care, Bad Homburg, Germany). Intervention group B will be managed with CVVHD using the high cut-off dialyzer Ultraflux EMiC2 (Fresenius Medical Care, Bad Homburg, Germany) and intervention group C using the high-flux filter Ultraflux AV1000S (Fresenius Medical Care, Bad Homburg, Germany) and in series the adsorber CytoSorb® (CytoSorbents Europe GmbH, Müggelseedamm 131, Berlin, Germany). The adsorber will be placed before dialyzer. Dialysis will be performed by multiFiltrate® (Fresenius Medical Care, Bad Homburg, Germany) in all groups.

In group A bicarbonate-buffered replacement fluid (multiBic® K4 or K2, Fresenius medical care, Bad Homburg, Germany) and in group B and C dialysate (CiCa® dialysate K4 or K2, Fresenius medical care, Bad Homburg, Germany) will applied.

The preferred anticoagulation of the extracorporeal circuit is regional citrate anticoagulation in group B and C. In group A systemic anticoagulation is required. The decision will be made by the attending intensivist in accordance with the recommendation of KDIGO.^12^
In group A systemic anticoagulation with heparin will be monitored twice daily by partial thromboplastin time (PTT) aiming 60s. First control of PTT will be after two hours of starting treatment. Heparin will be started with 18 IE/kg/h (ideal body weight) and adjusted during treatment period. If bleeding occur heparin should be reduced or stopped.
RCA of the extracorporeal circuit in group B and C will be monitored by measuring ionized postfilter calcium and guided by citrate supply (citrate: 136 mmol/l). An ionized postfilter calcium of 0.25-0.34 mmol/l will be targeted and at the start of treatment citrate flow will be set to 4.0 mmol citrate/l blood. To keep systemic ionized calcium stable between 1.12–1.20 mmol/l, a calcium chloride solution (calcium: 83 mmol/l) will be added to the extracorporeal circuit near the backflow to the patient. Flow will be started with 1.7 mmol Ca^2+^/l dialysate.
Total turnover rate (TTR) (dialysate or replacement fluid) will be calculated at 25 ml/kg ideal or adjusted body weight/h.^21^

Ideal body weight will be calculated using the Hamwi equation (for males: 48 kg for the first 152 cm + 1.1 kg for each additional cm; for females 45 kg for the first 152 cm + 0.9 kg for each additional cm). If the quotient of actual body weight divided by ideal body weight is more than 1.3,^22^ the adjusted body weight will be used for calculation of dialysate or replacement fluid flow (for males: (actual body weight-ideal body weight) * 0.38 + ideal body weight; for females: (actual body weight-ideal body weight) * 0.32 + ideal body weight).^23^

Blood flow (QB) will be the threefold of the dialysate flow and in the CVVH group blood flow will be just as 20% of the filtration fraction (QB=TTR/0.2).

Volume reduction will be defined by the attending intensivists considering patient`s requirements. According to the statement of the manufacturer, the maximum dialyzer lifespan will be limited to 72 hours and the maximum adsorber lifespan to 24 hours. After the first 24 hours the treatment should be continued, if myoglobin persists >4000 µg/l and ongoing reduction of renal function. In Group C (CVVHD with high-flux filter Fresenius Medical Care and adsorber) the adsorber has to be changed every 24h if myoglobin level persists > 4000ug/l and ongoing reduction of renal function. After 72h renal replacement therapy should be continued following physicians’ decision.

In group A (CVVH-postfilter with high-flux filter) and group B (CVVHD with HCO filter) blood samples will be collected before (pre) and after (post) dialyzer. In group C (CVVHD with high-flux filter and adsorber) blood samples will be taken before (pre) adsorber, between (int) adsorber and dialyzer and after (post) dialyzer.

**7.5. Endpoints**

**7.5.1. Primary endpoint:**

Marginal means of myoglobin clearance (CVVH, CVVHD-HCO und CVVHD-Cytosorb®)

**7.5.2. Secondary endpoints:**

- Clearance of other molecules with different molecule weight (creatinine, urea, beta-2 microglobulin, Il-6, albumin) after 1h, 6h, 12h and 24 hours
- Myoglobin clearance after 1h, 6h, 12h and 24 hours and in the course of time
- Lifetime of extracorporeal circuit (filter lifetime)
- 90 day outcome: Composite endpoint of patient dead, persistent loss of renal function requiring dialysis or persistent decline in GFR (>25% of baseline value)
- Clinical endpoints:
  - ICU length of stay (days)
  - ICU mortality
  - Hospital mortality
  - Mortality day 28
  - Mortality day 90
- Safety endpoints (Intervention period 72 hours):
  - Adverse events:
    - Hypocalcemia
    - Metabolic alkalosis
    - Citrate accumulation
    - Bleeding requiring blood transfusion
    - Heparin induced Thrombocytopenia (HIT)
    - Catheter malfunction
  - Severe adverse events:
    - Treatment-associated life-threatening complication
    - Dead of any cause during intervention

**7.6. Data collection:**

- Demographic and clinical data at time of initiating RRT:
  - renal function (creatinine, urea, diuresis)
  - admission diagnosis, indication for renal replacement therapy, Acute Physiology And Chronic Health Evaluation II (APACHE II), Sequential Organ Failure Assessment (SOFA), Simplified Acute Physiology Score II (SAPS II), mean arterial blood pressure, need for mechanical ventilation, need for vasopressor, sepsis, concomitant medication and pre-existing diseases.
- Laboratory and clinical data during the observation period (72 hours, daily monitoring):
  - creatinine, sodium, potassium, chloride, phosphate, calcium, magnesium, hemoglobin, hematocrit, platelet count, white blood cell count, albumin, pH, bicarbonate, base excess, lactate, mean arterial pressure, heart rate and oxygen saturation.
- Clinical follow up data:
  - Renal function after 90 days
  - Dialyzer lifetime (filter lifetime)
  - ICU and hospital mortality, length of ICU stay, 28-day mortality and 90-day mortality.
- Safety endpoints (Intervention period 72 hours):
  - Adverse events:
    - Hypocalcemia
    - Metabolic alkalosis
    - Citrate accumulation
    - Bleeding requiring blood transfusion
    - Heparin induced Thrombocytopenia (HIT)
    - Catheter malfunction
  - Severe adverse events:
    - Treatment-associated life-threatening complication
    - Dead of any cause during intervention

**7.7. Endpoints and calculations:**

Concentrations of myoglobin (17053 Dalton (Da), urea (60 Da), creatinine (113 Da), β2-microglobulin (11800 Da), interleukin 6 (IL-6, 26000 Da) and albumin (66470 Da) will be measured before (C_pre_) and after (C_post_) the dialyzer 1, 6, 12 and, 24 hours after initiating CRRT.
To avoid additional effect of hemoconcentration at the dialyzer, ultrafiltration will be set at zero ten minutes before drawing samples for laboratory analysis.
Plasma flow in the extracorporeal circuit (Qp_pre_) will be calculated using blood flow (Qb) of extracorporeal circuit and patient's hematocrit level (hct) at the time of sampling:

$${Qp}_{pre}(ml/min)=Qb\times((1-hct)\div100)$$

The substance-specific plasma clearance (Cl_p_) will be calculated at the sampling time points:

$${Cl}_{p}(ml/min)={Qp}_{pre}\times((C_{pre}-C_{post.})\div C_{pre})$$

The primary outcome parameter will be marginal means of myoglobin clearance. This will be calculated of myoglobin clearance after 1h, 6h, 12h, 24h (secondary endpoint). Other secondary outcome parameters will be plasma clearances of urea, creatinine, β2-microglobulin, interleukin 6 and albumin at the same time points.

Equality of plasma clearance (Cl_p.corr._) between the study groups relating to different TTR will be tested using the following formula:

$${Cl}_{p.corr.}={Cl}_{p}\times(\frac{{TTR}_{group B or C}}{{TTR}_{group A}})$$

In CVVH (control group), a sampling port is available only before instead of after the replacement fluid flows into the extracorporeal circuit. Therefore, the postfilter solute concentration (C_post_) has to be corrected to account for replacement fluid flow. For this purpose, postfilter plasma flow (Qp_post_) will be calculated subtracting filtration portion (FP; ml/min) from prefilter plasma flow (Qp_pre_):

$${Qp}_{post}(ml/min)={Qp}_{pre}-FP$$

The solute-specific concentration at the end of the extracorporeal circuit (C_post.corr._) will be adjusted using the ratio of plasma flow pre- and postfilter (Qp_post_/Qp_pre_) in CVVH group.

$$C_{post.corr.}(mmol/l)={(Qp}_{post}\div{QP}_{pre})\times C_{post}$$

**Laboratory analysis:**

Laboratory analyses will be performed using Cobas 8000 (Roche, Mannheim, Germany) according to the manufacturer´s instructions, immediately after sampling.

The following methods will be used:
- Urea: kinetic test with urease and glutamate dehydrogenase

- Creatinine: enzymatic method with creatinase

- β2-microglobulin: am c701 immunological test for turbidity

- Myoglobin: ElektroChemiLumineszenzImmunoAssay (ECLIA)

- IL-6: ECLIA

- Human albumin: color test with bromocresol green

**7.8. Statistical analysis:**

Because clinical data are not available, currently an exact sample size calculation is not possible. According to the character of the study as a pilot trial, the study questions are primarily explorative. That is, principally the data are analyzed exploratively: Categorical variables will be displayed as frequencies and percentages and median, minimum and maximum (or 25^th^ and 75^th^ quantile in square brackets) for continuous variables. Skew distributed data with outliers are logarithmic transformed before inclusion in linear models.

For the measurements of clearance of myoglobin and other molecules at the four time points, mixed linear models are applied. They model the correlation structure within the data and work in the presence of some missing values, too. By means of contrasts, the marginal mean for every method is estimated with 95% confidence interval (CI). The change of clearance over time is evaluated by other user-defined contrasts with CI. For the primary question, groups can be compared by contrast tests.

Group-separated point diagrams are appropriate for this sample size.

The Kaplan-Meier method will be applied to calculate and depict the survival function of the dialyzer lifetime.

All analyses will be performed using R, version 4 (R Core Team 2020, Vienna, A) and IBM SPSS, versions 26 (Minneapolis, USA). The significance level is defined 5% for two-tailed tests.

**8. References:**

1. Hoste EAJ, Bagshaw SM, Bellomo R, et al. Epidemiology of acute kidney injury in critically ill patients: the multinational AKI-EPI study. *Intensive Care Med*. 2015;41(8):1411-1423. doi:10.1007/s00134-015-3934-7

2. Uchino S, Kellum JA, Bellomo R, et al. Acute renal failure in critically ill patients: a multinational, multicenter study. *JAMA*. 2005;294(7):813-818. doi:10.1001/jama.294.7.813

3. Bosch X, Poch E, Grau JM. Rhabdomyolysis and acute kidney injury. *N Engl J Med*. 2009;361(1):62-72. doi:10.1056/NEJMra0801327

4. Panizo N, Rubio-Navarro A, Amaro-Villalobos JM, Egido J, Moreno JA. Molecular mechanisms and novel therapeutic approaches to rhabdomyolysis-induced acute kidney injury. *Kidney Blood Press Res*. 2015;40(5):520-532. doi:10.1159/000368528

5. Chavez LO, Leon M, Einav S, Varon J. Beyond muscle destruction: a systematic review of rhabdomyolysis for clinical practice. *Crit Care*. 2016;20(1):135. doi:10.1186/s13054-016-1314-5

6. El-Abdellati E, Eyselbergs M, Sirimsi H, et al. An observational study on rhabdomyolysis in the intensive care unit. exploring its risk factors and main complication: Acute kidney injury. *Ann Intensive Care*. 2013;3(1):1-8. doi:10.1186/2110-5820-3-8

7. Kasaoka S, Todani M, Kaneko T, et al. Peak value of blood myoglobin predicts acute renal failure induced by rhabdomyolysis. *J Crit Care*. 2010;25(4):601-604. doi:10.1016/j.jcrc.2010.04.002

8. Amyot SL, Leblanc M, Thibeault Y, Geadah D, Cardinal J. Myoglobin clearance and removal during continuous venovenous hemofiltration. *Intensive Care Med*. 1999;25(10):1169-1172. doi:10.1007/s001340051031

9. Zhang L, Kang Y, Fu P, et al. Myoglobin clearance by continuous venous-venous haemofiltration in rhabdomyolysis with acute kidney injury: A case series. *Injury*. 2012;43(5):619-623. doi:10.1016/j.injury.2010.08.031

10. Zeng X, Zhang L, Wu T, Fu P. Continuous renal replacement therapy (CRRT) for rhabdomyolysis. *Cochrane Database Syst Rev*. 2014;2014(6). doi:10.1002/14651858.CD008566.pub2

11. Liu C, Mao Z, Kang H, Hu J, Zhou F. Regional citrate versus heparin anticoagulation for continuous renal replacement therapy in critically ill patients: A meta-analysis with trial sequential analysis of randomized controlled trials. *Crit Care*. 2016;20(1):1-13. doi:10.1186/S13054-016-1299-0

12. KDIGO. Clinical Practice Guideline for Acute Kidney Injury (AKI). *Kidney Int Suppl*. 2012;2(1):4. doi:10.1038/kisup.2012.4

13. Heyne N, Guthoff M, Krieger J, Haap M, Häring H-U. High cut-off renal replacement therapy for removal of myoglobin in severe rhabdomyolysis and acute kidney injury: a case series. *Nephron Clin Pract*. 2012;121(3-4):c159-64. doi:10.1159/000343564

14. Weidhase L, Haussig E, Haussig S, Kaiser T, de Fallois J, Petros S. Middle molecule clearance with high cut-off dialyzer versus high-flux dialyzer using continuous veno-venous hemodialysis with regional citrate anticoagulation: A prospective randomized controlled trial. Isaka Y, ed. *PLoS One*. 2019;14(4):e0215823. doi:10.1371/journal.pone.0215823

15. Weidhase L, de Fallois J, Haußig E, Kaiser T, Mende M, Petros S. Myoglobin clearance with continuous veno-venous hemodialysis using high cutoff dialyzer versus continuous veno-venous hemodiafiltration using high-flux dialyzer: a prospective randomized controlled trial. *Crit Care*. 2020;24(1):644. doi:10.1186/s13054-020-03366-8

16. Hawchar F, László I, Öveges N, Trásy D, Ondrik Z, Molnar Z. Extracorporeal cytokine adsorption in septic shock: A proof of concept randomized, controlled pilot study. *J Crit Care*. 2019;49:172-178. doi:10.1016/j.jcrc.2018.11.003

17. Dimski T, Brandenburger T, Slowinski T, Kindgen-Milles D. Feasibility and safety of combined cytokine adsorption and continuous veno-venous hemodialysis with regional citrate anticoagulation in patients with septic shock. *Int J Artif Organs*. 2020;43(1):10-16. doi:10.1177/0391398819866459

18. Kousoulas L, Wittel U, Fichtner-Feigl S, Utzolino S. Hemoadsorption in a Case of Severe Septic Shock and Necrotizing Fasciitis Caused by Nontraumatic Renal Rupture due to Pyelonephritis with Obstructive Uropathy. *Case reports Crit care*. 2018;2018:5248901. doi:10.1155/2018/5248901

19. Dilken O, Ince C, van der Hoven B, Thijsse S, Ormskerk P, De Geus HRH. Successful Reduction of Creatine Kinase and Myoglobin Levels in Severe Rhabdomyolysis Using Extracorporeal Blood Purification (CytoSorb®). *Blood Purif*. 2020:743-747. doi:10.1159/000505899

20. Doig GS, Simpson F. Randomization and allocation concealment: a practical guide for researchers. *J Crit Care*. 2005;20(2):187-191. doi:10.1016/j.jcrc.2005.04.005

21. Fayad AI, Buamscha DG, Ciapponi A, Ai F, Dg B, Ciapponi A. Intensity of continuous renal replacement therapy for acute kidney injury ( Review ). *Cochrane Database Syst Rev*. 2016;(10):CD010613. doi:10.1002/14651858.CD010613.pub2.www.cochranelibrary.com

22. Glynn CC, Greene GW, Winkler MF, Albina JE. Predictive versus measured energy expenditure using limits-of-agreement analysis in hospitalized, obese patients. *JPEN J Parenter Enteral Nutr*. 1999;23(3):147-154. doi:10.1177/0148607199023003147

23. Krenitsky J. Adjusted Body Weight, Pro: Evidence to Support the Use of Adjusted Body Weight in Calculating Calorie Requirements. *Nutr Clin Pract*. 2005;20(4):468-473. doi:10.1177/0115426505020004468

**9. Abbrevations:**

AKI *acute kidney injury*

CI *confidence interval*

RRT *renal replacement therapy*

CK *creatinin kinase*

CVVH *continous veno-venous hemofiltration*

RCA  *regional citrate anticoagulation*

CVVHD *continous veno-venous hemodialysis*

HCO *high-cut off*

Il-6 Interleukin 6

AE *adverse event*

HIT heparin induced thrombocytopenia

BMI *body mass index*

SOFA *Sequential organ failure assessment*

SAPS *Simplified Acute Physiology Score*

**10. Correspondence:**

Jonathan de Fallois
Devision of Nephrology, Medical Clinic III
University Hospital Leipzig
Liebigstraße 18
04103 Leipzig

Germany
